# Dual-Structured Flexible Piezoelectric Film Energy Harvesters for Effectively Integrated Performance

**DOI:** 10.3390/s19061444

**Published:** 2019-03-24

**Authors:** Jae Hyun Han, Kwi-Il Park, Chang Kyu Jeong

**Affiliations:** 1 Department of Materials Science and Engineering, Korea Advanced Institute of Science and Technology, Daejeon 34141, Korea; jaehhan@kaist.ac.kr; 2 School of Materials Science and Engineering, Kyungpook National University, Daegu 41566, Korea; kipark@knu.ac.kr; 3 Division of Advanced Materials Engineering, Chonbuk National University, Jeonju, Jeonbuk 54896, Korea; 4 Hydrogen and Fuel Cell Research Center, Chonbuk National University, Jeonju, Jeonbuk 54896, Korea

**Keywords:** energy harvesting, piezoelectric, self-powered device, self-powered sensor, PZT film, flexible, laser lift-off, bending, mechanical neutral plane, finite element analysis

## Abstract

Improvement of energy harvesting performance from flexible thin film-based energy harvesters is essential to accomplish future self-powered electronics and sensor systems. In particular, the integration of harvesting signals should be established as a single device configuration without complicated device connections or expensive methodologies. In this research, we study the dual-film structures of the flexible PZT film energy harvester experimentally and theoretically to propose an effective principle for integrating energy harvesting signals. Laser lift-off (LLO) processes are used for fabrication because this is known as the most efficient technology for flexible high-performance energy harvesters. We develop two different device structures using the multistep LLO: a stacked structure and a double-faced (bimorph) structure. Although both structures are well demonstrated without serious material degradation, the stacked structure is not efficient for energy harvesting due to the ineffectively applied strain to the piezoelectric film in bending. This phenomenon stems from differences in position of mechanical neutral planes, which is investigated by finite element analysis and calculation. Finally, effectively integrated performance is achieved by a bimorph dual-film-structured flexible energy harvester. Our study will foster the development of various structures in flexible energy harvesters towards self-powered sensor applications with high efficiency.

## 1. Introduction

In recent years, energy harvesting technologies have drawn attention from many researchers hoping to establish self-powered sensors and Internet of Things (IoT) systems for future applications [1,2]. Among the various energy sources in our surroundings, mechanical energy sources are highly promising for individual energy harvesting devices because mechanical energy is pervasive (e.g., machinery vibration, body activity, biomechanical movement, natural stimulation, etc.) but often wasted unwittingly [3,4]. In terms of this aspect of mechanical energy sources, the energy harvesting technology largely means the field of mechanical energy harvesting [1,2]. Moreover, mechanical energy harvesting devices can be also used as mechanical sensors without external power sources [5,6]. Therefore, energy harvesting materials and devices are important concepts for a new era of sensor applications.

Although some principles have been developed to convert mechanical energy to electrical energy, piezoelectric materials and devices have still been considered as prospective energy harvesting technology owing to the simple device structures and environmental robustness regardless of wear, humidity, and serious heaviness [4,7]. In addition, piezoelectric energy harvesters can be fabricated as flexible electronics for next-generation electronic systems using piezoelectric ceramics as well as polymers on flexible plastic substrates [8,9,10]. For example, piezoelectric energy harvesters can be applied to tiny vibration-based energy harvesting and sensor applications polyvinylidene fluoride or ceramics [11,12,13,14].

In fact, triboelectric energy harvesting, which is based on the coupling between tribelectrification and electrostatic induction, is also a promising technology for self-powered sensor devices. Triboelectric energy harvesters can generate very high voltage signals [15,16,17]. In contrast, they are weak during high humidity and very long duration owing to the mechanism of contact electrification [18,19]. Moreover, piezoelectric devices can be more easily fabricated in a flexible configuration [19]. Therefore, triboelectric and piezoelectric energy harvesters should be utilized according to proper and complementary applications.

Various technologies have been reported to fabricate piezoelectric ceramics-based flexible energy harvesters, such as the stamping transfer method, polymer-hybrid composites, single-crystalline ceramic lamination, and so on [20,21,22,23,24,25]. However, these approaches suffer from instable fabrication, non-reliable performance, insufficient output, and/or very high cost. In contrast, the recently developed technology for flexible ceramic film energy harvesters using laser lift-off (LLO) processes is a highly plausible approach due to low-cost materials, excellent scalability, and high output density [26,27,28,29]. Furthermore, the LLO process is already commercialized in the field of optoelectronics and display devices, which guarantees the processing reliability and the compatibility with conventional fabrications of electronics [27,30,31]. Nonetheless, the LLO fabrication-based energy harvester should be developed for enhancing the current or charge density performance because the current level of the previously reported device is much weaker than that of single-crystalline laminated flexible energy harvesters [25,32]. Although the energy harvesting signals (indicating the generated output voltage and current) can increase by connecting multiple devices to each other, it is a very complicated configuration in terms of practical energy or sensor applications. Additionally, for sensors based on energy harvesters and self-powered applications, the generated power output is very important. In the field of piezoceramic thin/thick film-based flexible energy harvesters in bending mode, state-of-the-art power output in instantaneous bending motions has been reported from 160 μW to 200 μW [29,33].

Even though the performance enhancement of the LLO-based flexible thin film energy harvesters has been studied using the quantitative simulations and the textured polycrystalline piezoelectric films resulting from modifying basal mother wafers [33], the crystallographic approach requires a special, expensive wafer (e.g., MgO, LaAlO_3_, etc.), while the processing yield becomes low. Therefore, the original LLO process for the flexible thin film energy harvesters should be revisited to improve the device performance and stability. Similar to the technology of multilayer ceramic capacitor (MLCC), flexible thin film energy harvesters can also be enhanced by using layered piezoelectric ceramic films on a single area of plastic substrate. However, there has not yet been a systematic investigation into layered flexible piezoelectric thin film energy harvesters for effectively enhanced and integrated energy harvesting performance.

In this study, we demonstrate two different types of flexible thin film energy harvesters as prototypes of multilayered (dual-film-structured) piezoelectric generators fabricated by the reported stable LLO process. One type is a stacked dual-film structure, while the other is a double-faced (bimorph) dual-film structure. Because both devices are fabricated by the guaranteed LLO process and piezoelectric ceramics, the intrinsic material properties are very reasonable and reliable. Nevertheless, the performance of the two structures is highly different due to the differences in mechanical strain and generated piezopotential of piezoceramic films. The stacked dual-film energy harvester cannot generate effective energy harvesting performance, while the bimorph energy harvester presents good energy harvesting performance and reasonably integrated energy harvesting output for performance enhancement. The important discrepancy originates from the location of mechanical neutral plane for applied effective strain in piezoelectric active layers. Thus, it is noted that the device performance cannot simply be improved by increasing the number of piezoelectric active layers in a single flexible film energy harvester. Our investigation provides an appropriate design and perspective for multilayered piezoelectric film-based flexible energy harvesters towards performance-enhanced self-powered mechanical sensor systems.

## 2. Materials and Methods

### 2.1. Materials

We used PbZr_0.52_Ti_0.48_O_3_ (PZT) for the piezoelectric ceramics of the flexible film energy harvester because it is commonly used for various piezoelectric applications. In particular, the 52/48 composition is well known as the most effective piezoelectric composition of the PZT material system, called the morphotropic phase boundary (MPB) [14,33,34]. The PZT sol-gel solution of 0.4 M was purchased from Quintess Co. Ltd. (Incheon, Korea). The sapphire wafer was purchased from Hi-Solar Co. Inc. (Gwangju, Korea). The flexible polyethylene terephthalate (PET) substrate (125 μm in thickness) was purchased from Sigma-Aldrich (St. Louis, MO, USA). The polyurethane (PU)-based curable adhesive was bought from Norland Products Inc. (Cranbury, NJ, USA). The SU-8 photoresist (PR) solution for the encapsulation layer was purchased from MicroChem Co. (Westborough, MA, USA).

It should be noted that PZT is the most widely used piezoelectric material for various applications because it has highly piezoelectric properties and relatively easy processes. However, it contains lead (Pb), which is very harmful to biological systems. Because of the risk of lead-based poisoning, a variety of lead-free piezoelectric materials have been developed, especially for biomedical device applications [35,36].

### 2.2. Fabrication of the Stacked Dual-Film-Structured Flexible Energy Harvester

**Figure 1:**
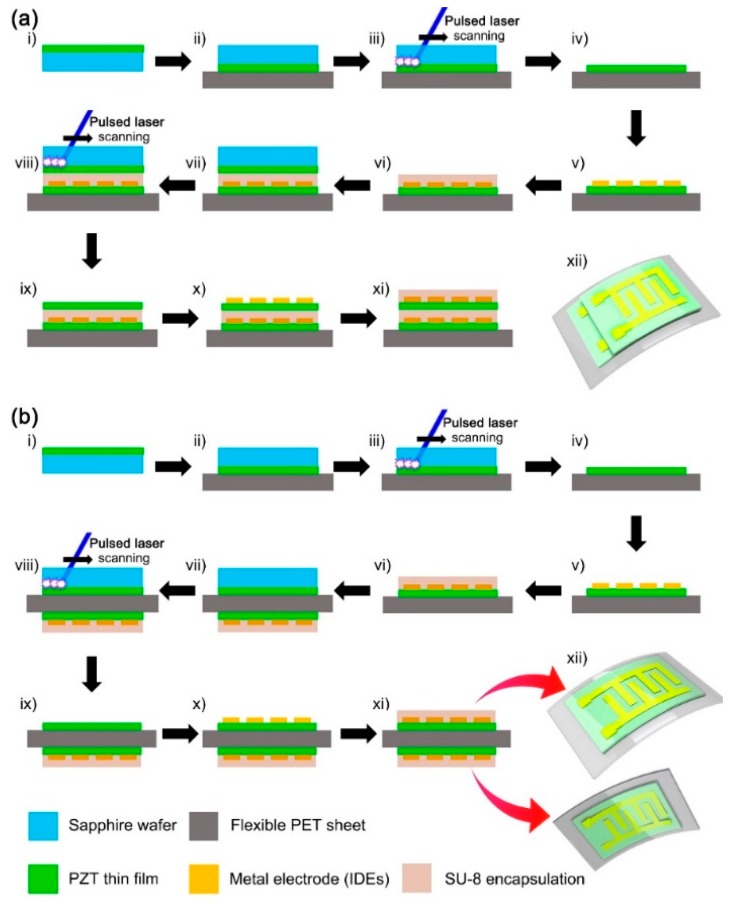
Fabrication steps of (**a**) the stacked dual-film-structured flexible energy harvester and (**b**) the double-faced dual-film-structured flexible energy harvester as cross-sectional schematics (i‒xi). The final architectures are illustrated as three-dimensional schematics (xii).

The fabrication steps of the stacked device are depicted as cross-sectional schematics in Figure 1a (i to xii). The PZT sol-gel solution was spin-casted on the sapphire wafer, and annealed at 450 °C in rapid thermal annealing (RTA) processes for 10 min for pyrolysis and crystallization. The single deposition can make a PZT thin film of ~100 nm in thickness. The spin coating was repeated several times to achieve a PZT film of ~2 μm in thickness, which was the optimized condition for the flexible PZT thin film energy harvesters [26,27,33]. The deposited PZT film was finally crystallized at 650 °C in a furnace for 1 h for complete crystallization (i). The surface of deposited PZT film was attached on the flexible PET sheet substrate using PU-based adhesive (ii). Then, the backside of sapphire wafer was irradiated by the XeCl pulsed excimer laser (wavelength of 308 nm, shot area of 625 μm × 625 μm) with programmed scanning (iii). Because the backside of the sapphire wafer was also well polished (no surface scattering) and the band gap energy of sapphire (~9 eV) is much higher than the photonic energy of XeCl laser (4.03 eV), the laser beam can definitely penetrate the body of the sapphire wafer. On the contrary, the PZT film should fully absorb the laser photonic energy since the bandgap energy of PZT (~3.2 eV) is smaller than the laser energy. Thus, the PZT should be microscopically decomposed or ablated at the interface with the sapphire wafer, and completely transferred onto the flexible PET sheet (iv). This is the first PZT layer of dual-film-structured flexible energy harvesting devices. Interdigitated electrodes (IDEs) were patterned on the flexible PZT film using Cr/Au evaporator deposition (10 nm/100 nm) and conventional lithography. The geometrical width and gap of IDEs were 100 μm and 100 μm, respectively (v). IDEs is the effective electrode type for bending-mode piezoelectric energy harvesters because it can use the d_33_ piezoelectric mode when the device is bent [26]. The dimension of IDE width and gap was optimized for the poling process to avoid electric breakdown with effective electric field. In contrast, metal‒insulator‒metal (MIM) structured electrodes use the d_31_ mode in bending. For the PZT system, d_33_ is higher than d_31_. Note that the active area of a PZT thin film with electrodes is about 1.2 cm × 1.2 cm. It should be mentioned that the simple geometry or area of IDEs can be modified in comparison with previous reports [26,27,28,29,33]. The encapsulation layer was subsequently formed by coating the SU-8 PR resin (~5 μm in thickness) except contact holes for wiring and measuring (vi). The encapsulation layer also acted as a buffer basement for the second PZT layer transfer of a stacking-structured energy harvester. The PZT-deposited sapphire wafer was again attached on the encapsulation layer (vii). The LLO process was conducted one more time to establish the second transferred PZT layer (viii). Then, the PZT layer was transferred onto the as-fabricated first-layered device structure (ix). It should be noted that the first PZT layer could be easily destroyed if there was no buffer layer, presumably due to undesirable thermal and mechanical shocks. On the second PZT layer, the same IDEs and the stabilizing encapsulation layer were formed subsequently (x and xi). The final device architecture of a stacked dual-film-structured energy harvester is illustrated as a three-dimensional schematic (xii). After wiring, each PZT film was poled by the 100 kV·cm^−1^ through the IDEs.

### 2.3. Fabrication of the Bimorph Dual-Film-Structured Flexible Energy Harvester

The fabrication steps of the bimorph device are also described as cross-sectional schematics in Figure 1b (i to xii). For the as-fabricated first-layered device structure with only the first PZT layer, the process was exactly the same as the fabrication of stacked device in the Section 2.2 (i to vi). After that, the second PZT layer deposited on another sapphire wafer was attached onto the backside of the flexible PET sheet substrate of the as-fabricated one-layered PZT device (vii). The XeCl excimer laser was irradiated onto the backside of the sapphire wafer again (viii). The second PZT layer was transferred from the sapphire to the backside of the PET substrate, so double-faced PZT films were achieved on the single PET substrate (ix). The IDEs and SU-8 encapsulation were also fabricated on the second PZT layer of PET backside using the same deposition and lithography (x and xi). The complete device architecture of bimorph dual-film-structured energy harvester is illustrated in the three-dimensional schematic (xii). This device was also poled by the 100 kV·cm^−1^ through the IDEs, but each PZT film should be poled by opposite polarity because the stress applied to each film must be opposite when the device is bent in measurement (tension and compression, respectively). Both devices were measured by a Keithley 6514 electrometer (Tektronix Inc., Beaverton, OR, USA) and a customized bending machine stage based on a linear motor (Jeil Optical System Co., Incheon, Korea), as shown in Supplementary Figure S1. The generated voltage and current were measured at open-circuit and short-circuit conditions, respectively, except for the load resistance experiment.

## 3. Results and Discussion

### 3.1. Analyses of PZT Films As-Deposited and Transferred by Multiple Laser Lift-Off Processes

**Figure 2:**
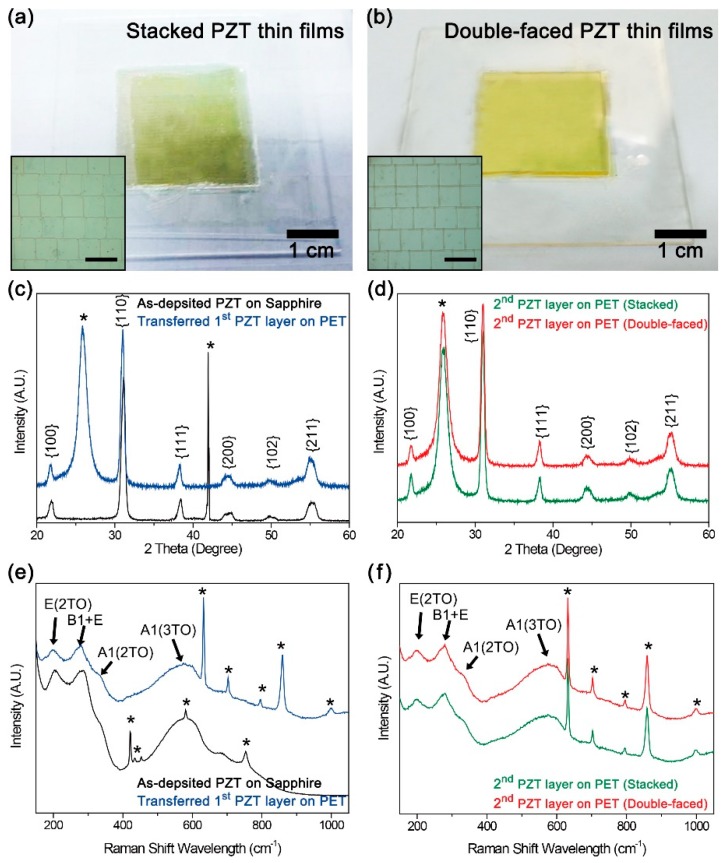
Photographs of (**a**) stacked dual-structured PZT films and (**b**) double-faced dual-structured PZT films transferred onto the single flexible PET sheet. Insets: optical microscopy images of secondly transferred PZT films of each dual-film structure. Scale bars: 1 mm. XRD patterns of (**c**) the as-deposited PZT film and first transferred PZT film and (**d**) the second transferred PZT films of stacked and bimorph device structures. The asterisks indicate the peaks resulting from the sapphire wafer or the PET sheet. Raman spectra of (**e**) the as-deposited PZT film and first transferred PZT film and (**f**) the second transferred PZT films of stacked and bimorph device structures. The asterisks indicate the signals stemming from the sapphire wafer or the PET substrate.

Figure 2a,b shows photographs of stacked dual-structured PZT films and bimorph PZT films, which are multiple-transferred onto the single flexible PET sheet substrate. Both structures were well established by the optimum processes. The first LLO of PZT film has already been well defined by the previously reported strict procedure [26,27,33]. However, the second LLO step of next PZT films onto the as-fabricated device can cause material failure due to undesirable effects such as abnormal thermal gradients, mechanical deteriorations, interfacial roughness, and so forth.

For the stacked device, failure (e.g., crack) easily occurs when the second PZT film is detached from the sapphire wafer after LLO because it should be aided by mechanical force applied by tweezers, which can induce the fracture of the underlying first PZT film. This problem can be solved by a method in which the second PZT film is transferred onto the first layer device so as not to overlap completely, as shown in Figure 2a. The bimorph device is also generally hard to fabricate using LLO, because the backsideof the PET sheet is not flat due to the already-established first device part, which may hinder the laser focusing for backside LLO. Adopting a semi-cured polydimethylsiloxane (PDMS) coated glass substrate as a handle wafer during the second LLO can alleviate this problem because the first device part could be conformally buried into the PDMS buffer on the glass substrate. Thus, the backside of the sheet can be relatively flat for the laser focusing. Throughout our optimized multistep LLO processes for dual-structured PZT films, there are no serious visual problems, as presented by the optical microscopy images (insets of Figure 2a,b). Note that the square patterns on the transferred PZT result from the shape of the pulsed laser beam [26,27,33].

To investigate the material characteristics of PZT films in detail, we compared the X-ray diffraction (XRD) patterns and Raman spectra of the as-deposited PZT film on the sapphire wafer, the first transferred PZT film, and the second transferred PZT films in the two different dual-film structures. Figure 2c shows the XRD patterns of the PZT thin film on the sapphire wafer deposited by the sol-gel processing and the first transferred PZT film on the flexible PET substrate by the initial LLO. As expected, the crystallized PZT film of MPB composition was defined by the sol-gel deposition, and well maintained on the PET sheet after the LLO process [27]. In addition, the secondly transferred PZT films by subsequent LLO processes also present same perovskite MPB crystal structures, as shown in Figure 2d. In both stacked and bimorph structures, the second PZT films are stably transferred without degradation. It means that there is no crystallographic and compositional modulation in the transferred PZT films after multistep LLO processes because the duration time of pulsed excimer laser is very short (~30 ns), localizing the laser heating only within the interface [27].

Raman spectroscopy also shows the well-retained MPB phase structure of PZT films during the multistep LLO processes, as presented by Figure 2e,f. Raman spectroscopy was performed to analyze the phase of the PZT films using a 514.5 nm Ar^+^ laser source as an excitation source at ambient temperature. The bands of the Raman shifts around 205, 275, 330, and 591 cm^−1^ correspond to the typical property of perovskite MPB phase of PZT [26,33]. The above bands correspond to E(2TO), B1+E, A1(2TO) and A1(3TO) modes, respectively [37]. Note that there may be also more complicated Raman mode bands of the perovskite phase, but they do not appear clearly in the spectrum of thin film materials due to weak signals and substrate effects [26]. The Raman characteristics of the secondly transferred PZT films are also the same as those of the as-deposited and the first transferred PZT films.

### 3.2. Energy Harvesting Performance of Two Different Dual-Film-Structured Flexible Energy Harvesters

A stacked structure is the first way to enhance and integrate the energy harvesting performance of PZT thin-film energy harvesters in a single device because various integrated devices exist in vertically stacked structures like the MLCC. Therefore, our stacked dual-film-structured flexible film energy harvester was investigated first.

**Figure 3:**
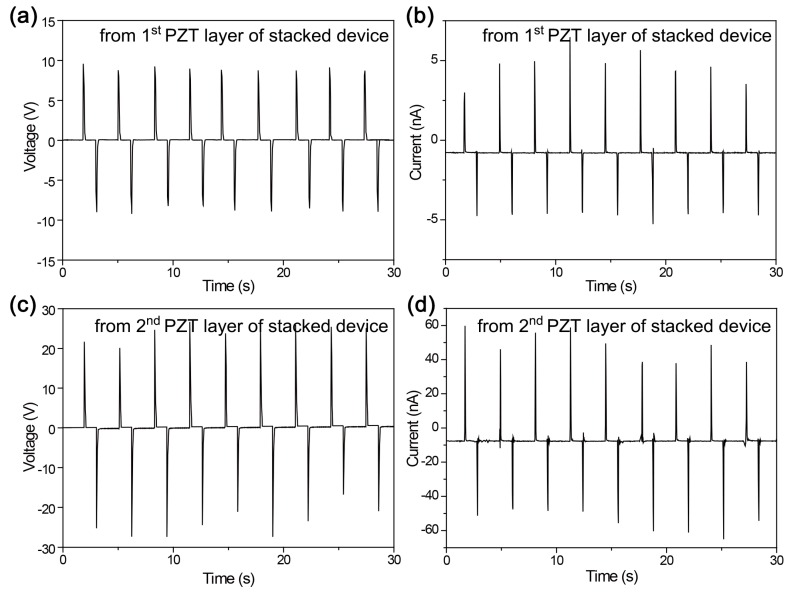
(**a**) Voltage and (**b**) current signals generated by the first PZT layer of the stacked dual-film-structured flexible energy harvester. (**c**) Voltage and (**d**) current signals produced by the second PZT layer of the stacked dual-film-structured flexible device.

One might expect that each PZT layer can generate high-performance energy harvesting signals and the performance can be merged for higher voltage and current signals. As shown in Figure 3a,b, however, the first PZT layer produces excessively low energy harvesting signals, i.e., ~9 V of voltage and ~4 nA of current, in the bending stimulation. This energy harvesting performance is useless compared to the previous general flexible PZT thin film energy harvester made by LLO processes, which generated ~140 V of voltage and ~1 μA. Although the signals from the second PZT layer (~25 V and ~50 nA) are better than those of the first layer (Figure 3c,d), they are still much poorer than from the general flexible PZT film generator. If there is no consideration of the mechanics of flexible devices, this phenomenon looks weird because the basic structure, fabrication, and measurement conditions (bending radius of ~1.6 cm) are identical to our previous reports for flexible single-layered PZT film energy harvesters.

**Figure 4:**
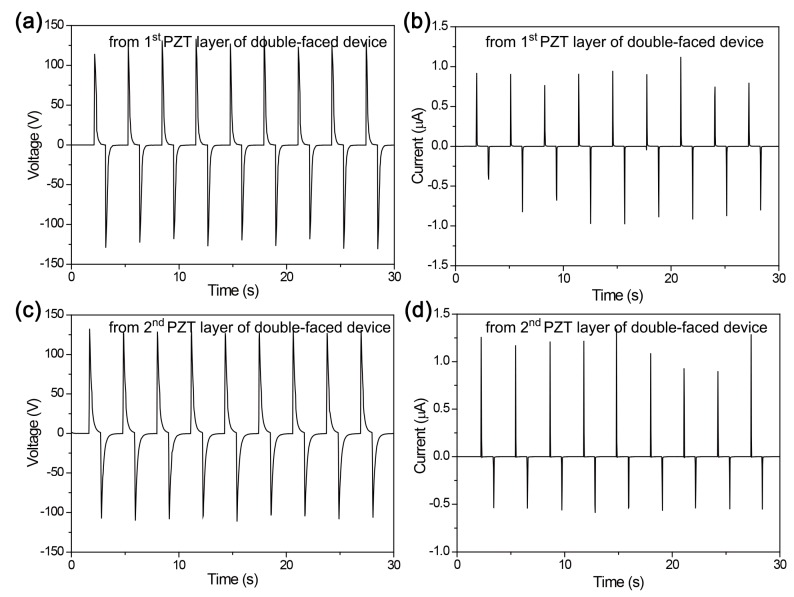
(**a**) Voltage and (**b**) current signals generated by the first (top-sided) PZT layer of the bimorph dual-film-structured flexible energy harvester. (**c**) Voltage and (**d**) current signals produced by the second (bottom-sided) PZT layer of the bimorph dual-film-structured flexible device.

In contrast, the bimorph dual-film-structured energy harvester generates the expected and high-performance signals in the same bending condition [26,27]. As presented by Figure 4, both the first (top-sided) and the second (bottom-sided) PZT layers converted the convex and concave bending strain into the electrical peaks, respectively. The voltage level is up to 140 V and the current peak is up to 1.3 μA. Note that the peak amplitude of the bottom-side PZT layer is slightly asymmetric presumably because it is governed by compressive stress and strain, different from the general measurements of other flexible energy harvesters as well as the top-side PZT layer under tensile stress and strain.

### 3.3. Theoretical Simulations and Final Selection for Integrated Performance

**Figure 5:**
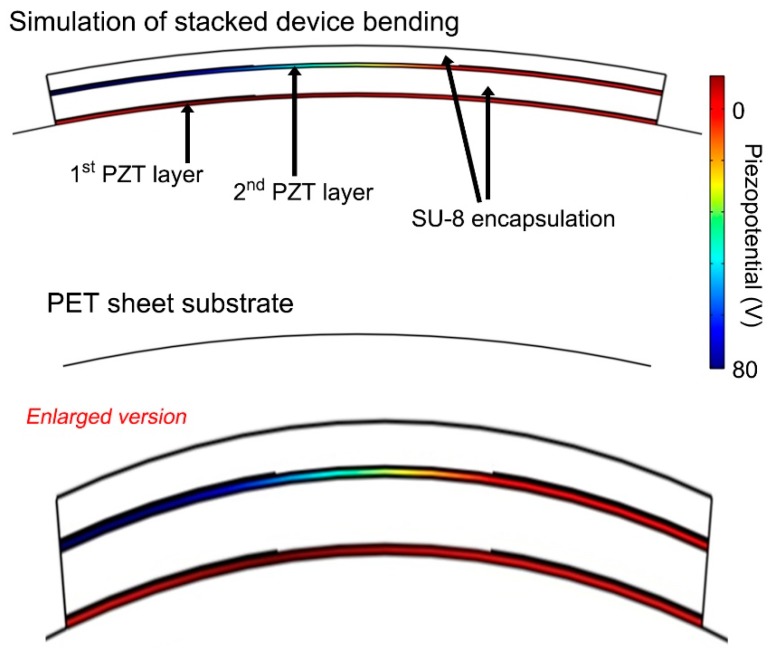
FEA theoretical simulation of the flexible stacked dual-film-structured PZT film energy harvester, showing the applied strain in the bending stimulation. The scale is identical to one pair of IDE fingers, except enlarged figures, which are for clear visualization.

**Figure 6:**
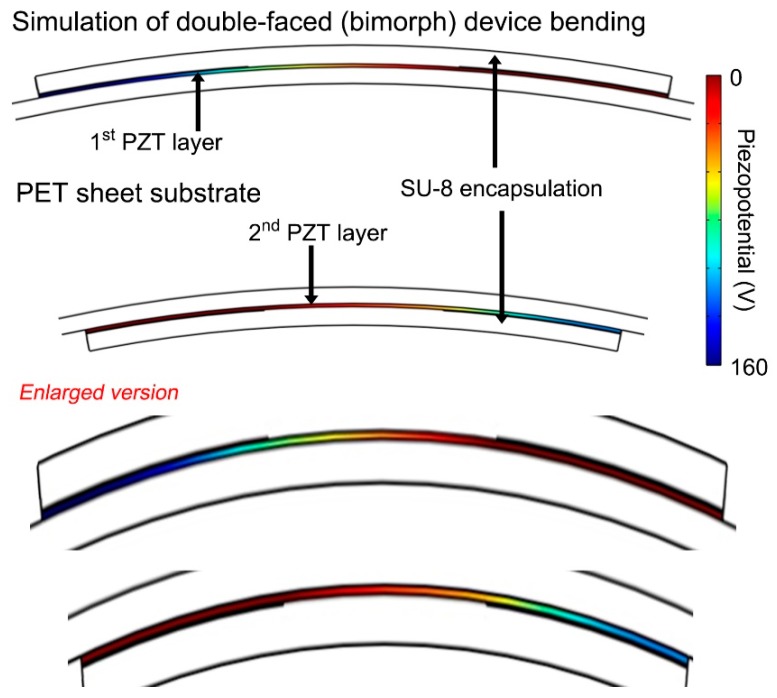
FEA theoretical simulation of the flexible bimorph dual-film-structured PZT thin film energy harvester, showing the applied strain in the bending stimulation. The scale is identical to one pair of IDE fingers, except enlarged figures, which are for clear visualization.

To investigate the difference between the performance of the stacked dual-film-structured flexible energy harvester and the bimorph dual-film-structured flexible device, we performed the finite element analysis (FEA) simulations using COMSOL Multiphysics computation program (Figure 5 and Figure 6). All material parameters were based on the confirmed values and previously reported simulations. As shown in Figure 5, interestingly, the simulated piezoelectric potential of the first PZT layer of the stacked structure is relatively very low (~20 V), while that of the second layer is relatively decent (~70 V).

This serious discrepancy should be analyzed by the mechanical neutrality in the flexible device configuration. The position of the mechanical neutral plane in a layered device structure can be described as below Equation (1):

(1) $$h_{\mathrm{neutral}}=\frac{\sum_{i=1}^{N}\left\{\bar{Y_{i}}t_{i}\left(\sum_{j=1}^{i}t_{j}-\frac{t_{i}}{2}\right)\right\}}{\sum_{i-1}^{N}\bar{Y_{i}}t_{i}},$$

where *N* is the total number of layers, *t_i_* is the thickness of the *i*th layer (from the top), and is $\bar{Y{\;}_{i}}=\;Y_{i}/(1-\upsilon_{i}^{2})$ is defined as the effective Young’s modulus (*Y_i_* ≡ absolute Young’s modulus and υ*_i_* ≡ Poisson’s ratio of the *i*th layer) [38]. For the stacked dual-film-structured flexible PZT thin film energy harvester, the layer structure is the sequence of SU-8 encapsulation, second PZT thin film, SU-8 encapsulation, first PZT thin film, and PET sheet substrate (from the top). The mechanical parameters and thicknesses are (1) *Y*_SU-8_ = 4.02 GPa, υ_SU-8_ = 0.22, and *t*_SU-8_ = 5 μm; (2) *Y*_PZT_ = 62.5 GPa, υ_PZT_ = 0.35, and *t*_PZT_ = 2 μm; and (3) *Y*_PET_ = 3 GPa, υ_PET_ = 0.4, and *t*_PET_ = 125 μm.

The mechanical neutral plane is ~48 μm below the top surface. Therefore, the distance between the mechanical neutral plane and the middle of first PZT film is about 35 μm. The applied bending strain in the PZT layer is calculated by Equation (2):

(2) $$\varepsilon =\frac{\delta }{r},$$

where *r* is the bending radius and *δ* is the distance from the mechanical neutral plane [38]. The calculated strain in the first PZT thin film at bending radius of 1.6 cm is about 0.217%. On the other hand, the distance between the mechanical neutral plane and the middle of second PZT layer is about 42 μm, indicating the strain applied to the second layer is about 0.260% in the same mechanical stimulation. The much lower energy harvesting performance in both PZT layers of the stacked dual-film-structured device is because these much smaller applied strain in bending stimulations than that of the general one-layer PZT thin film energy harvester. Thus, this phenomenon results from the mechanics of layered structures in flexible device configuration.

In contrast, the generated piezopotential of each PZT layer of the bimorph structure is generally high (~160 V), as shown in Figure 6. This is in accordance with the trend of the experimental measurement. Note that the polarity of piezopotential between the first and second PZT layers is opposite because they are under tensile and compressive stress, respectively. It can be merged and integrated by the proper poling process and wire connection.

The computational result can also be analyzed by Equations (1) and (2) for the mechanical neutral plane and the actually applied strain. According to Equation (1), the location of the mechanical neutral plane in a layered device structure is ~69.5 μm below the top surface. Hence, the distance between the mechanical neutral plane and the middle of each PZT film is about 63.5 μm because the bimorph dual-film-structured device has the symmetric cross-sectional configuration. This means that the applied bending strain to the PZT layers of bimorph device is about 0.394% in the same bending stimulation, slightly higher than that of the general one-layer PZT thin film energy harvester [26,33].

**Figure 7:**
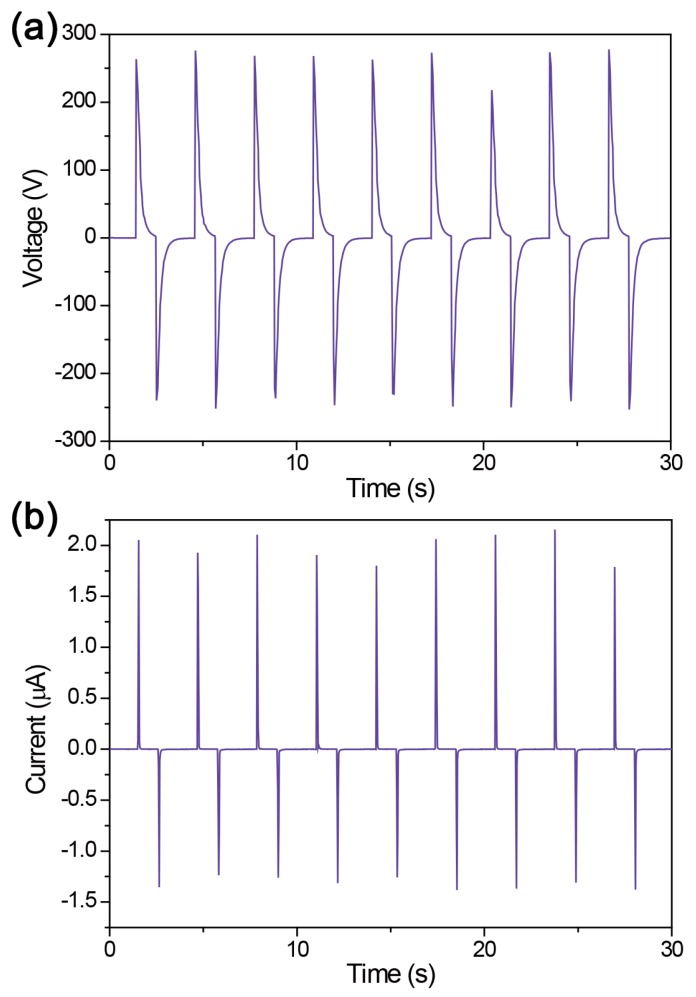
Integrated energy harvesting signals of the flexible bimorph PZT film energy harvester.

As declared previously, the performance enhancement of energy harvesting by signal integration in a single device is very important without any complicated multiple device arrangements or complex material modifications, in order to facilitate more rapid technological growth for commercialization of the advantages of LLO technology-based flexible thin film energy harvesters with high performance. Therefore, we integrated efficiently the generated signals from both PZT layers of the double-faced dual-film-structured flexible energy harvester for effectively enhanced performance. As presented by Figure 7, the energy harvesting signals are well correspondingly merged and integrated, showing the voltage up to ~280 V in series and the current up to ~2.2 μA in parallel. Consequently, it is reasonable that the bimorph structure is adoptable and rational for the effectively integrated performance of a flexible PZT thin film energy harvester in a single device. It is easily guaranteed by the performance of single-faced (uni-morph) structured device with the same condition (Figure S2). The uni-morph energy harvester generates ~120 V and 0.8 μA, which is smaller even than the single part of bimorph device. This is presumably due to the applied strain in the uni-morph device is smaller [26,33]. All generated energy harvesting signals are also briefly summarized in Table S1 to show our results at a glance. Note that the integrated output signals are not the exact sum of each single-faced device, which can be considered a minor non-linear effect. This phenomenon also commonly occurs in the widely used linear superposition test for energy harvesters. It is presumably due to the parasitic capacitance in circuits of multiple-connected electrodes [39,40]. Moreover, this is more visible in the current output because the current signals are more irregular than voltage output generally in the piezoelectric energy harvesters with bending motions. Using the bimorph integrated flexible energy harvester, we also measured the instantaneous power output according to the external circuit load resistance, as shown in Figure S3. The maximum power, ~180 μW, was presented at ~100 MΩ, which is a similar matching resistance in IDE-based flexible energy harvesters [26]. According to the area of electrode pairs, the maximum instantaneous power density is about 250 μW·cm^−2^.

## 4. Conclusions

In summary, we have demonstrated two different dual-film-structured flexible thin film energy harvesters for effectively integrated energy harvesting performance: a stacked structure and a bimorph structure. The LLO process-based fabrication has been selected for the basic energy harvesting device because it was confirmed to realize flexible high-performance harvesting unit devices. However, there have still been only a few ways to enhance the performance and/or integrate the energy level of the flexible film energy harvesters, which is highly significant for the future progress of commercialization, sensor applications, and self-powered electronics based on piezoelectric device principles. Multiple device connection is not useful due to the complicated features. Material modification approach was too expensive to achieve the improvement of the flexible energy harvesting unit. Therefore, we have suggested integrated structures of PZT film devices in a single area, like the approaches of MLCC. Both stacked and bimorph structures of dual PZT thin films fabricated by multistep LLO processes are well established on the flexible PET sheet substrate. However, the stacked dual-film-structured flexible energy harvester cannot generate effective energy harvesting signals, while the bimorph structure shows the general performance of PZT thin film energy harvesters. Based on the theoretical simulations and calculations, it is well discussed that the phenomena is due to the ineffective position of mechanical neutral plane in the stacked dual-films structure. Therefore, we propose the bimorph structure to effectively integrate and improve the performance of flexible PZT thin-film energy harvester as a single device configuration. As we expected, the energy harvesting performance of the double-faced device are the voltage up to ~280 V and the current up to ~2.2 μA, which indicated the good performance enhancement and integration in the bending deformation. Because the piezoelectric energy device and technology can be directly associated with various sensor systems [41,42,43,44], our experimental and theoretical demonstration for performance improvement of flexible thin film energy harvesters using the intuitive and simple approach will promote more rapidly the practical developments of flexible piezoelectric energy harvesting devices for future self-powered and self-sufficient sensor systems.

## Supplementary Materials

The following are available online at https://www.mdpi.com/1424-8220/19/6/1444/s1, Figure S1: Photographs of bending machine stage and clamped devices when the energy harvesting signals are measured, Figure S2: Generated (a) voltage and (b) current signals by a single-faced (uni-morph) energy harvesting device, Figure S3: Instantaneous power generated by the bimorph-integrated device according to external load resistance. The power is calculated by voltage divided by resistance square at the certain load resistance, Table S1: Generated voltage and current signals by each PZT layer of the two different devices, uni-morph device, and the final successful integrated devices by the bimorph (double-faced) device. These values are maximum values of each unit.
