# Supplementary material for: Dual-Structured Flexible Piezoelectric Film Energy Harvesters for Effectively Integrated Performance
Source: Sensors (Basel). 2019 Mar 24;19(6):1444. doi: 10.3390/s19061444 (PMC6470648; doi:10.3390/s19061444)
Supplement: Supplementary file 1 [file sensors-19-01444-s001.pdf]

Supplementary Materials

Dual-Structured Flexible Piezoelectric Film Energy Harvesters for Effectively Integrated Performance

Jae Hyun Han [1], Kwi-Il Park [2] and Chang Kyu Jeong [3,4,*]

[1] Department of Materials Science and Engineering, Korea Advanced Institute of Science and Technology, Daejeon 34141, Korea; jaehhan@kaist.ac.kr
[2] School of Materials Science and Engineering, Kyungpook National University, Daegu 41566, Korea; kipark@knu.ac.kr
[3] Division of Advanced Materials Engineering, Chonbuk National University, Jeonju, Jeonbuk 54896, Korea
[4] Hydrogen and Fuel Cell Research Center, Chonbuk National University, Jeonju, Jeonbuk 54896, Korea
* Correspondence: ckyu@jbnu.ac.kr; Tel.: +82-63-270-2380

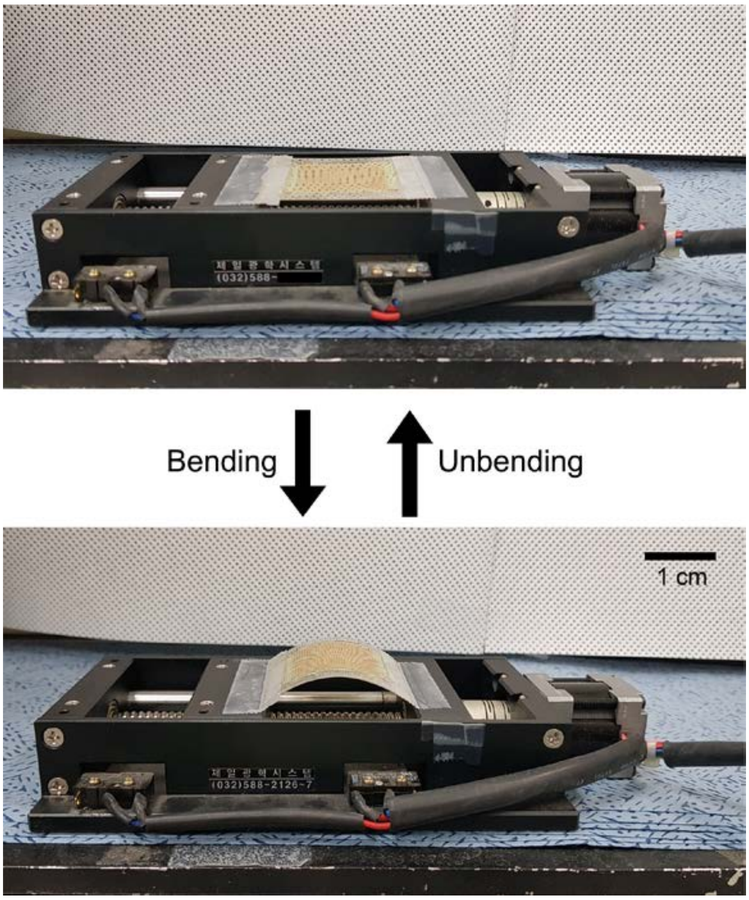

Supplementary Figure S1. Photographs of bending machine stage and clamped devices when the energy harvesting signals are measured.

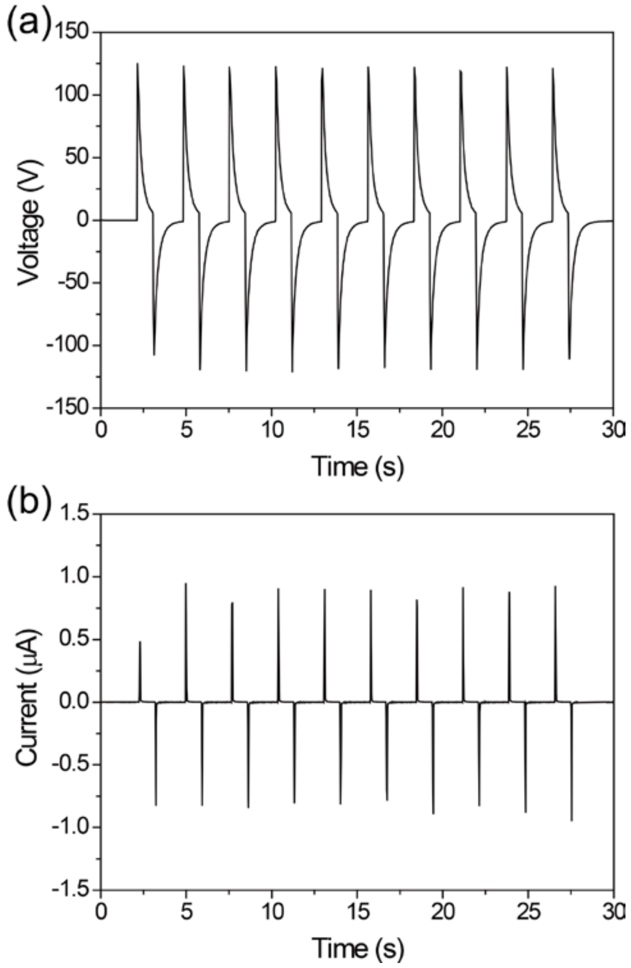

Supplementary Figure S2. Generated (a) voltage and (b) current signals by a single-faced (uni-morph) energy harvesting device.

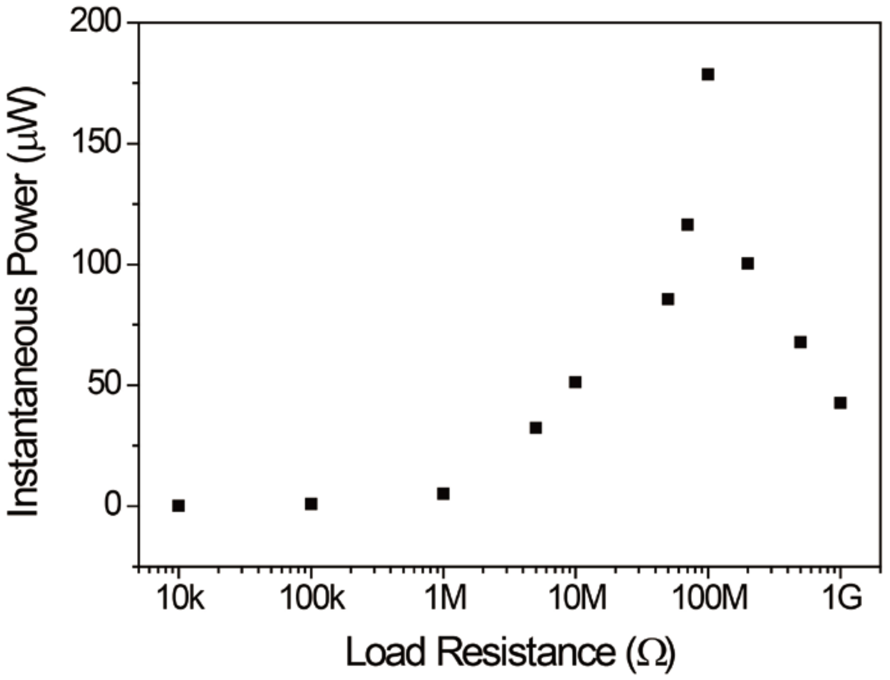

Supplementary Figure S3. Instantaneous power generated by the bimorph-integrated device according to external load resistance. The power is calculated by voltage divided by resistance square at the certain load resistance.

Type of Layers	Voltage	Current
1st layer of stacked device	~9 V	~4 nA
2nd layer of stacked device	~25 V	~50 nA
1st layer of double-faced (bimorph) device	~140 V	~1.1 μA
2nd layer of double-faced (bimorph) device	~140 V	~1.3 μA
Previous single-faced (uni-morph) device	~120 V	~0.8 μA
Successful integration (bimorph)	~280 V	~2.2 μA

Supplementary Table S1. Generated voltage and current signals by each PZT layer of the two different devices, uni-morph device, and the final successful integrated devices by the bimorph (double-faced) device. These values are maximum values of each unit.
